# Internal regulation between constitutively expressed T cell co-inhibitory receptors BTLA and CD5 and tolerance in recent thymic emigrants

**DOI:** 10.1098/rsob.240178

**Published:** 2024-10-30

**Authors:** Adeolu O. Adegoke, Govindarajan Thangavelu, Ting-Fang Chou, Marcos I. Petersen, Kiyokazu Kakugawa, Julia F. May, Kevin Joannou, Qingyang Wang, Kristofor K. Ellestad, Louis Boon, Peter A. Bretscher, Hilde Cheroutre, Mitchell Kronenberg, Troy A. Baldwin, Colin C. Anderson

**Affiliations:** ^1^ Department of Surgery, University of Alberta, Edmonton, AB, Canada; ^2^ Alberta Diabetes and Transplant Institutes, University of Alberta, Edmonton, AB, Canada; ^3^ La Jolla Institute for Immunology, La Jolla, CA 92037, USA; ^4^ Department of Medical Microbiology and Immunology, University of Alberta, Edmonton, AB, Canada; ^5^ Laboratory for Immune Crosstalk, RIKEN Center for Integrative Medical Sciences, 1-7-22 Suehiro, Tsurumi-ku, Yokohama 230-0045, Japan; ^6^ JJP Biologics, Warsaw, Poland; ^7^ Department of Microbiology and Immunology, College of Medicine, University of Saskatchewan, Saskatoon, SK, Canada; ^8^ Department of Molecular Biology, University of California San Diego, La Jolla, CA 92093, USA

**Keywords:** T cells, autoimmunity, BTLA, CD5, recent thymic emigrants, tolerance

## Abstract

Immunologic self-tolerance involves signals from co-inhibitory receptors. Several T cell co-inhibitors, including PD-1, are expressed upon activation, whereas CD5 and BTLA are expressed constitutively. The relationship between constitutively expressed co-inhibitors and when they are needed is unknown. Deletion of *Btla* demonstrated BTLA regulates CD5 expression. Loss of BTLA signals, but not signalling by its ligand, HVEM, leads to increased CD5 expression. Higher CD5 expression set during thymic selection is associated with increased self-recognition, suggesting that BTLA might be needed early to establish self-tolerance. We found that BTLA and PD-1 were needed post-thymic selection in recent thymic emigrants (RTE). RTE lacking BTLA caused a CD4 T cell and MHC class II dependent multi-organ autoimmune disease. Together, our findings identify a negative regulatory pathway between two constitutively expressed co-inhibitors, calibrating their expression. Expression of constitutive and induced co-inhibitory receptors is needed early to establish tolerance in the periphery for RTE.

## 1. Introduction

To generate repertoire diversity, developing T cells in the thymus express receptors generated from random recombination of TCR gene segments [1]. The outcome of this stochastic TCR gene segment rearrangement is the generation of T cells bearing receptors that recognize foreign antigens or self-antigens as agonists, with the latter potentially promoting autoimmune diseases, although varying degrees of beneficial autoreactivity are present in regulatory T cells (Treg) and innate-like T cells, such as natural killer T cells (iNKT cells). Positive selection of T cells creates an additional potential danger, as conventional CD4^+^ and CD8αβ^+^ T cells are positively selected based on an ability to recognize a self-peptide–MHC complex. This process provides the benefit of an ability to optimally recognize non-self peptides that are very near to self in terms of sequence [2]. Hence, a critical function of the immune system is to discriminate self from non-self, failure of which will result in detrimental autoimmunity. Thus, most T cells that bind self-peptide–MHC with sufficient affinity need to be made tolerant, and all T cells need to be maintained in a state where they perceive low-affinity interactions with self-peptide–MHC ligand (e.g. the positively selecting peptide) as a ‘tonic’ signal rather than an agonist signal. Although T cell activation and tonic signalling are determined by the interaction of TCR with specific antigenic peptide–MHC complexes, the functional outcome of the T cell response is strongly influenced by co-stimulatory and co-inhibitory signals [3,4]. Such co-inhibitory receptors, like programmed cell death protein-1 (PD-1) and cytotoxic T lymphocyte-associated antigen-4 (CTLA-4), are absent from naive T cells and are upregulated upon activation [5,6]. By contrast, there are a small number of co-inhibitory receptors expressed constitutively by naive T cells, including CD5, B and T lymphocyte attenuator (BTLA), and V-domain immunoglobulin suppressor of T cell activation (VISTA) [7–10] .

CD5 is a co-inhibitory receptor constitutively expressed on post-selected thymocytes, mature T cells and a subset of B cells (B1a cells) [7,10–13]. CD5 is expressed as a 67 kDa type I transmembrane glycoprotein (gp), which belongs to the highly conserved superfamily of protein receptors known as the scavenger receptor cysteine rich (SRCR) superfamily [14]. The proposed ligands for CD5 include: CD72 [15], IL-6 [16,17], gp40-80 [18–20], gp150 [21], gp200 [22], IgV_H_ framework region [23] and CD5 itself [24]; however, their physiological relevance and relative importance in interacting with CD5 remains an active area of investigation. CD5 also appears to function without its extracellular domain, questioning whether a ligand for CD5 is relevant. During T cell activation, CD5 is rapidly recruited and co-localizes with the TCR/CD3 complex at the immune synapse, dampening downstream TCR signals [25,26].

BTLA (CD272) is a negative regulator of antigen receptors on B and T cells, dendritic cells, macrophages and NK cells [8,27–29]. The BTLA cytoplasmic region contains both ITIM and ITSM motifs and a Grb2 recognition motif [8]. Following T cell activation and the interaction of BTLA with its ligand, the herpesvirus entry mediator (HVEM or CD270), the tyrosine residues in ITIM and ITSM of BTLA are phosphorylated and then recruit SH2-containing tyrosine phosphatase 1 (SHP-1) and SHP-2 phosphatases to dampen TCR signalling [28,30]. A T cell can express both BTLA and HVEM, and HVEM interactions with BTLA between cells, in *trans*, or *cis* interactions in which one cell expresses both binding partners, are inhibitory [31]. Consistent with its co-inhibitory function, mice lacking BTLA develop systemic autoimmune disease and multi-organ lymphocytic infiltration [32]; they also have increased frequencies of T follicular helper cells in Peyer’s patches and increased IgG and IgA, the latter affecting the homeostasis of gut bacteria [33]. By contrast, *Cd5^−/−^* mice remain relatively healthy, even in late life [34]. Although constitutively expressed BTLA and CD5 on T cells have unique non-redundant roles, the relationship between these co-inhibitors is not well defined. It was recently reported that CD5 expression levels increased in SHP-1 knockout T cells relative to WT T cells [35]. Since BTLA and CD5 exert their inhibitory function through lymphocyte activation-induced recruitment of SHP-1 to their cytoplasmic tail tyrosine residues, these two co-inhibitors might have some overlapping or counter-regulatory functions. In addition, CD5 expression is set during T cell development in the thymus and finely adjusted throughout the life of the T cell [36,37], with CD5 surface expression correlating directly with the avidity or signalling intensity of TCR–self-peptide MHC interaction [36,38]. CD5 is expressed most highly on T cells in the thymus [36,39] and on recent thymic emigrants in the periphery [40]. CD5 levels are modified by the autoimmune disease associated H-2^g7^ haplotype [41]. Given the above, CD5 levels might identify stages where the self-reactive potential of the T cell repertoire is greatest. Herein, we explored the relationship between these co-inhibitors, and found that BTLA broadly controls CD5 expression levels from early in T cell ontogeny and that both BTLA and PD-1 are needed to establish peripheral tolerance at the recent thymic emigrant stage.

## 2. Results

### 2.1. Negative regulation of CD5 expression by BTLA

**Figure 1.**
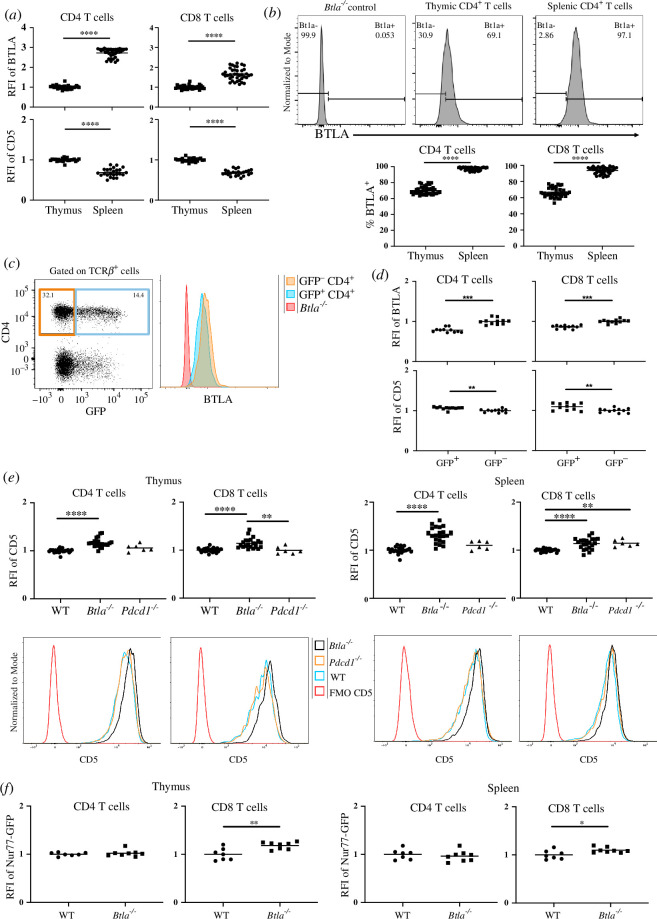
BTLA expression in the thymus and spleen is inversely related to CD5 expression. (*a*) Relative fluorescent intensities (RFIs) of BTLA (upper row) or CD5 (lower row) in CD4 SP T cells (left column) and CD8 SP T cells (right column) in the thymus and spleen (BTLA, *n* = 39; CD5, *n* = 24). To calculate the RFI of BTLA or CD5, mean fluorescence intensity (MFI) data were normalized to the average MFI of BTLA or CD5 of the thymic SP T cells in each individual experiment. (*b*) Representative histograms showing the proportion of BTLA^+^ CD4 SP T cells (upper row) in the thymus and spleen. Lower row shows the proportion of BTLA^+^ CD4 SP T cells (lower left) and BTLA^+^ CD8 SP T cells (lower right) in the thymus and spleen (*n* = 39). (*c*) Representative flow cytometry dot plot (left) and histogram (right) of the indicated markers in splenic TCRβ^+^ cells from 7 to 10 week old B6.*Rag2p^GFP^* mice. (*d*) RFIs of BTLA (top panels) and CD5 (lower panels) on mature (GFP^−^) or newly generated (GFP^+^) SP T cells in the spleen (*n* = 11). Data are normalized to the average BTLA MFI or CD5 MFI of the GFP negative splenic SP T cells in each individual experiment. (*e*) CD5 expression on CD4 SP T cells and CD8 SP T cells from thymus (left) or spleen (right) and their corresponding representative histograms (lower rows) from WT (*n* = 24; B6.*Foxp3^GFP^* and B6.*Nur77^GFP^*), *Btla^−/−^* (*n* = 22; B6.*Foxp3^GFP^ Btla^−/−^* and B6.*Nur77^GFP^ Btla^−/−^*) and *Pdcd1*^−/−^ (*n* = 6; B6.*Foxp3^EGFP^ Pdcd1*^−/−^) mice. (*f*) Nur77-GFP expression. Dots indicate individual mice from six to nine independent experiments (*a,b*,*e*) or two independent experiments (*c,d*,*f*). *p < 0.05, **p < 0.01, ***p < 0.001, ****p < 0.0001.

To examine the relationship between CD5 and BTLA, we assessed CD5 and BTLA expression in the steady state in both TCRβ^hi^ single positive (SP) T cells in the thymus and splenic T cells from 7- to 10-week-old C57BL/6 mice (electronic supplementary material, figure S1A). Consistent with earlier studies [36,39], thymic SP T cells expressed higher levels of CD5 relative to splenic SP T cells (figure 1*a*). By contrast, expression of BTLA was lower in the thymic SP T cells relative to splenic SP T cells (figure 1*a*). Analysis of the proportion of BTLA^+^ cells among thymic and splenic SP T cells also revealed a reduced frequency of BTLA^+^ SP T cells in the thymus relative to the spleen (figure 1*b*). This inverse relationship between BTLA and CD5 was apparent when comparing expression in central versus peripheral lymphoid organs but not within each tissue individually; T cells with the highest BTLA expression in each tissue did not have reduced CD5 expression (electronic supplementary material, figure S1B). These data suggested that BTLA and CD5 have opposite trajectories of expression as T cells mature.

A small fraction of splenic T cells are recent thymic emigrants (RTE). We hypothesized that these newly generated T cells would also express lower levels of BTLA relative to their established or more mature T cell counterparts. To examine this, we used the *Rag2p^GFP^* mice, where GFP expression is restricted to thymocytes, RTE and newly generated B cells [42,43]. Analysis of BTLA expression comparing the GFP^+^ (RTE) and GFP^−^ (mature) T cells in the spleen revealed that BTLA expression was significantly lower in RTE relative to their mature T cell counterparts (figure 1*c*,*d*); BTLA expression on T cells also increased as GFP expression decreased in both the spleen and thymus (electronic supplementary material, figure S2). By contrast, CD5 expression was significantly higher on RTE (figure 1*d*). Collectively, these data indicated an inverse relationship between CD5 and BTLA expression as T cells mature.

To determine if there was a causal relationship between BTLA expression and CD5 expression, we compared CD5 expression between polyclonal WT and co-inhibitor BTLA-deficient T cells in the periphery. We analysed PD-1 in parallel, because we showed earlier that it plays a role in the establishment of tolerance in newly generated T cells [44,45]. The expression of CD5 was significantly higher in *Btla^−/−^* splenic CD4 and CD8 T cells relative to their WT and PD-1-deficient (*Pdcd1^−/−^*) counterparts; the overall increase in CD5 mean fluorescence intensity (MFI) of *Btla^−/−^* T cells was due to a reduction in CD5 low cells (figure 1*e*). Congenital absence of the inducible co-inhibitor PD-1 resulted in significantly increased CD5 expression in splenic CD8 T cells (figure 1*e*). PD-1 deficiency did not alter BTLA expression (electronic supplementary material, figure S3A). To assess whether the elevated CD5 expression in *Btla^−/−^* splenic T cells originated from the thymus or preferentially increased in the periphery, we compared CD5 expression between the WT, *Pdcd1^−/−^* and *Btla^−/−^* thymic TCRβ^hi^ SP cells. These cells in the *Btla^−/−^* but not *Pdcd1^−/−^* mice expressed higher CD5 than their WT counterparts (figure 1*e*). A possible explanation is that BTLA is expressed on a much higher percentage of thymocytes than PD-1. WT, *Btla^−/−^* and *Pdcd1^−/−^* T cells expressed a similar level of CD5 in DN and DP populations (electronic supplementary material, figure S3B,C), indicating that CD5 expression in the *Btla^−/−^* T cells increased only post-thymic selection. Although only a small fraction of thymic or splenic T cells expressed the inducible co-inhibitor PD-1, BTLA deficiency also increased its expression (electronic supplementary material, figure S3D).

Treg cells make up a relatively small proportion of the bulk CD4^+^ T cell population, but the preferential expression of higher levels of CD5 in Treg cells [46] may skew the CD5 expression levels in the *Btla^−/−^* CD4^+^ T cells. Therefore, we analysed Treg numbers [47] and CD5 expression in splenic and thymic TCRβ^+^ CD4^+^ Foxp3^+^ (Treg) or TCRβ^+^ CD4^+^ Foxp3^−^ (non-Treg) cells of WT and *Btla^−/−^* mice (electronic supplementary material, figure S4). CD5 expression was significantly higher in both Treg and non-Treg cells of the *Btla^−/−^* mice in the spleen and thymus (electronic supplementary material, figure S4C,D).

Since CD5 expression is correlated with the signalling strength of TCR:self-antigen (self-pMHC) interactions [36,38] and Nur77 serves as a specific reporter of antigen receptor signalling in murine and human T and B cells [48–54], we hypothesized that Nur77^GFP^ expression would be enhanced in *Btla^−/−^* T cells. We generated *Btla^−/−^ Nur77^GFP^* mice and compared their GFP expression levels in the splenic and thymic T cells to that in BTLA sufficient *Nur77^GFP^* mice. Thymic and splenic *Btla*^*−/−*^ CD8 T cells expressed higher levels of GFP relative to the WT CD8 T cells (figure 1*f*). By contrast, GFP expression in the thymic and splenic *Btla^−/−^* CD4 T cells was not significantly different from their WT CD4 T cell counterparts (figure 1*f*). Since Nur77^GFP^ expression in B cells also correlates to the B cell receptor affinity for antigen [52], we compared GFP expression levels in B cells from *Btla^−/−^ Nur77^GFP^* mice and WT *Nur77^GFP^* mice and found no significant difference between the two groups (electronic supplementary material, figure S4E). Together, these data suggest that BTLA expression directly or indirectly determined the level of CD5 expression across the major conventional T cell subsets, while having a more limited effect on Nur77 expression.

### 2.2. BTLA regulates CD5 expression in adult mice

**Figure 2.**
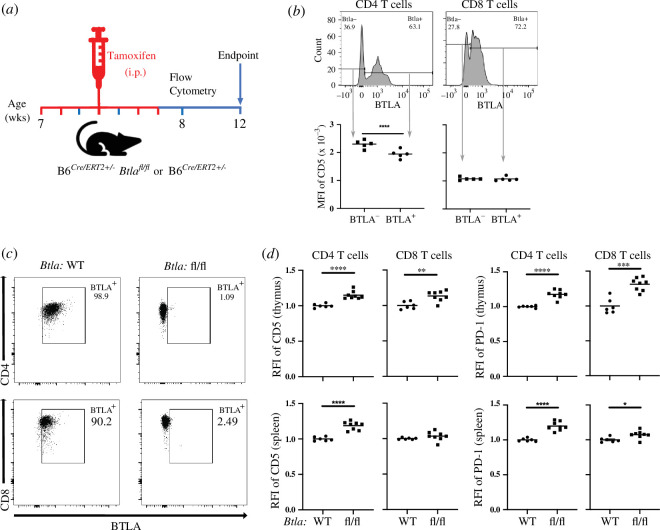
Loss of BTLA in adult mice leads to increased T cell CD5 and PD1 expression. (*a*) Adult B6*^Cre/ERT2+/−^* or B6*^Cre/ERT2+/−^ Btla^fl/fl^* mice received five doses of tamoxifen on days 0, 1, 3, 5 and 6 (highlighted in red). (*b*) Representative histograms (top) of BTLA expression and the MFI of CD5 (bottom) of BTLA^+^ and BTLA^−^ cells in the CD4 gated (left) and CD8 gated (right) T cells in the peripheral blood at one week post-tamoxifen. (*c*) Representative dot plots of BTLA expression in the splenic CD4 SP T cells (top) and CD8 T cells (bottom) in the B6*^Cre/ERT2+/−^* mice (left) and B6*^Cre/ERT2+/−^ Btla^fl/fl^* mice (right) at two weeks post-tamoxifen. (*d*) RFI of CD5 and PD-1 in the thymic (top row) CD4 and CD8 or splenic (bottom row) CD4 and CD8 T cells of B6*^Cre/ERT2+/−^* (WT) and B6*^Cre/ERT2+/−^ Btla^fl/fl^* (fl/fl) mice at four weeks post-tamoxifen. Dots indicate individual mice from two independent experiments. *p < 0.05, **p < 0.01, ****p < 0.0001.

**Figure 3.**
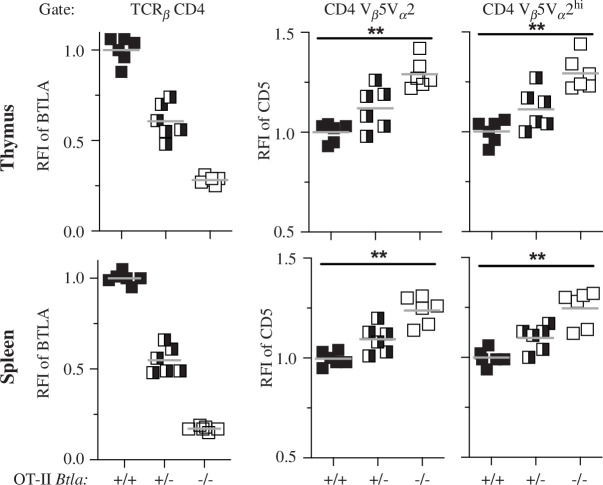
BTLA deficiency increases CD5 levels in TCR transgenic OT-II CD4 T cells in thymus and spleen. RFI of BTLA gated on TCRβ^+^ CD4^+^ SP cells (left) and RFI of CD5 in all CD4^+^ TCR-Vβ5Vα2^+^ cells (middle) and RFI of CD5 in CD4^+^ SP cells expressing high levels TCR-Vβ5Vα2 (right; gate included only the top approximately 50% of TCR-Vβ5Vα2 expressing cells). Analysis of T cells from the thymus (top) and spleen (bottom) of OT-II.*Btla^+/+^* (WT OT-II; *n* = 6), OT-II.*Btla^+/^*^−^ (*n* = 6) and OT-II.*Btla^−/−^* mice (*n* = 6). The grey line is the mean of the RFI. CD5 expression on OT-II.*Btla^+/+^* T cells was significantly lower than on BTLA-deficient (*Btla^−/−^*) OT-II T cells, **p < 0.01.

We determined whether the lack of BTLA from early in T cell ontogeny, as in the case of the germline *Btla* knockout mice, is important for producing heightened T cell CD5 expression. For example, the lack of BTLA during the neonatal period might alter the TCR repertoire when it is first generated, indirectly affecting CD5 levels. We therefore assessed the effect of deleting *Btla* in young adult mice by crossing *B6.Btla^fl/fl^* mice to a tamoxifen-inducible Cre recombinase expressing strain, *B6^Cre/ERT2^*, to generate *B6^Cre/ERT2+/−^ Btla^fl/fl^* mice (electronic supplementary material, figure S5). We injected adult 7-week-old *B6^Cre/ERT2+/−^ Btla^fl/fl^* and WT control *B6^Cre/ERT2+/−^* mice intraperitoneally with tamoxifen (figure 2*a*). At one-week post-tamoxifen injection only about a third of T cells circulating in the blood had lost BTLA expression. However, the BTLA-deficient fraction of CD4 T cells had significantly heightened CD5 expression (figure 2*b*). By two weeks post-tamoxifen, *B6^Cre/ERT2+/−^* splenic CD4 and CD8 SP T cells, were 97 ± 2% and 94 ± 4% positive for BTLA, respectively, while *B6^Cre/ERT2+^Btla^fl/fl^* T cells were reduced to 2 ± 1% and 3 ± 2% positive for BTLA (figure 2*c*). CD5 expression on thymic and splenic T cells had not yet significantly changed at this time point relative to WT controls (data not shown). By four weeks post-tamoxifen, CD5 and PD-1 expression in the thymic and splenic CD4 T cells and thymic CD8 T cells of *B6^Cre/ERT2+/−^ Btla^fl/fl^* mice had increased relative to their *B6^Cre/ERT2+/−^* counterparts (figure 2*d*). These data indicated that the impact of BTLA deficiency on CD5 and PD-1 expression is not specific to T cell ontogeny early in life; rather, it can be instigated acutely in adulthood. The rapid increase in CD5 expression on peripheral circulating CD4 T cells, induced by BTLA deletion, suggested that BTLA may be capable of regulating CD5 expression after the early generation of the TCR repertoire and in mature, peripheral T cells. Consistent with the possibility that BTLA regulates CD5 independent of TCR repertoire changes, we did not detect any major differences in the repertoire of sorted thymic CD4 SP T cells of WT and *Btla^−/−^* mice assessed by single cell RNA sequencing (electronic supplementary material, figure S6); however, these findings do not rule out potential subtle differences in the repertoire of *Btla^−/−^* mice. Therefore, we tested the effect of BTLA on CD5 expression when the TCR repertoire is fixed. We crossed the *Btla* gene knockout strain to OT-II [55] and OT-I [56] TCR transgenic mice. Complete BTLA deficiency (OT-II.*Btla^−/−^*) led to heightened CD5 expression in OT-II T cells in the periphery and the thymus (figure 3). OT-II T cells that expressed high levels of the transgenic TCR lacked endogenous TCR expression [57]. The heightened CD5 in BTLA-deficient OT-II T cells was evident also in those CD4 SP thymocytes and splenocytes expressing high levels of the transgenic TCR (Vβ5Vα2), indicating that endogenous TCR expression and TCR repertoire changes were not responsible for the altered CD5 expression (figure 3). In a preliminary analysis of OT-I CD8 T cells that naturally expressed very high levels of CD5 [58], BTLA deficiency further increased CD5 expression (electronic supplementary material, figure S7).

### 2.3. HVEM signals do not net set the level of CD5 expression

The only known ligand for BTLA is HVEM, a member of the tumour necrosis factor receptor superfamily (TNFRSF) and therefore is designated as TNFRSF14. HVEM also functions as a signalling receptor, recruiting tumour necrosis factor receptor-associated factor 2 (TRAF2) and activating NK-κβ RelA, to co-stimulate T cells [59]. HVEM signalling is also involved in survival of memory CD4 [60] and memory CD8 T cells [61,62]. Given the potential for bi-directional signalling, regulation of CD5 and PD-1 expression by BTLA could be due to BTLA signals, HVEM-mediated signals or both. We therefore examined CD5 and PD-1 expression in germline HVEM knockout mice (*Hvem^−^*^/−^) and mice engineered to express a ‘tail mutant’ HVEM with the intact ectodomain but lacking the normal sequence of the intracytoplasmic domain and therefore incapable of activating NF-κB (*Hvem^tm/tm^*).

Using CRISPR-Cas9 technology, we generated an *Hvem* mutant lacking exon 7 with a cytoplasmic tail sequence created by splicing exon 6 to exon 8 (electronic supplementary material, figure S8A). This sequence lacks the proline-x-glutamate amino acid motif likely required for TRAF binding and HVEM signalling [63]. We validated the lack of HVEM signalling by the mutant HVEM *in vitro* in transfected cells using cells expressing HVEM ligands and interacting with cells expressing the tail mutant HVEM with an NF-κB reporter as a readout (electronic supplementary material, figure S8B). *In vivo*, the tail mutant HVEM had lower surface expression than either WT mice or *Hvem^WT/tm^* hemizygous mice (electronic supplementary material, figure S8C). Despite this, we validated that the reduced amount of surface HVEM was sufficient *in vivo* in a liver injury model induced by activating iNKT cells with the potent agonist α-galactosylceramide (αGalCer). Previous studies show that either BTLA or CD160 serve as attenuators of αGalCer-mediated acute hepatic injury [64–66], and this occurs via engagement of HVEM [67]. To test the importance of HVEM signalling function in this hepatic injury model, we co-housed littermate WT control, *Hvem^tm/+^* hemizygous, and *Hvem^tm/tm^* mice that were injected with αGalCer and analysed 24 h later. *Hvem^tm/tm^* mice displayed a similar serum alanine aminotransferase (ALT) level compared with WT or hemizygous mice (electronic supplementary material, figure S8D), indicating HVEM acts as a ligand for signalling inhibitory receptors in this model.

**Figure 4.**
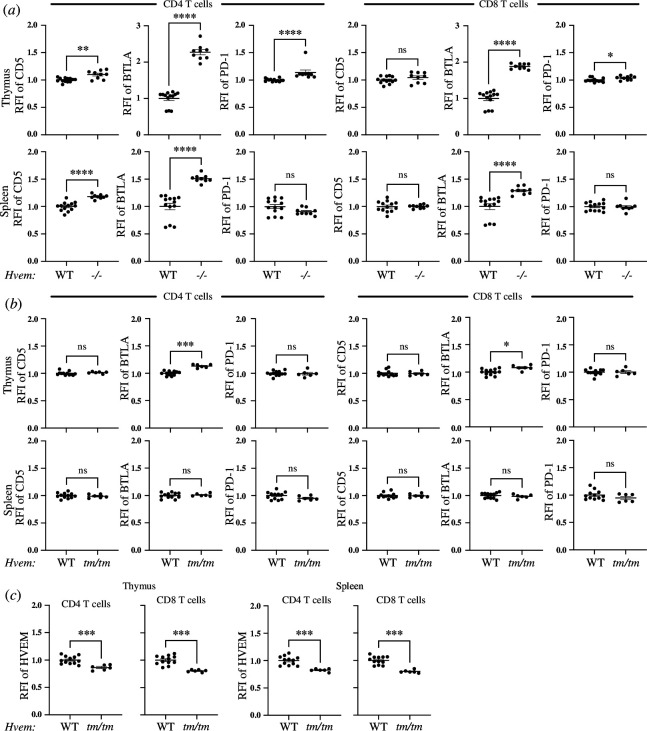
HVEM dependent control of CD5 and PD-1 expression from early in CD4 T cell ontogeny does not require HVEM signalling. Thymocytes and splenocytes from WT and *Hvem^−/−^* (*a*) or *Hvem^tm/tm^* mice (*b*,*c*), gated on SP CD4 and SP CD8 T cells, were examined by flow cytometry for expression of CD5, BTLA, PD-1 and HVEM. *p <0.05, **p < 0.01, ***p < 0.001, ****p < 0.0001.

We found that CD4 SP T cells from *Hvem^−^*^/−^ mice expressed higher CD5 in the thymus and spleen as well as higher PD-1 in the thymus (figure 4*a*). This elevated CD5 and PD-1 expression occurred despite thymic and splenic CD4 and CD8 SP T cells also having heightened BTLA expression in *Hvem^−^*^/−^ mice, in agreement with a previous report [68]. The heightened BTLA expression, however, could not compensate for the lack of the only known BTLA binding partner. By contrast, despite having lower levels of HVEM than either WT mice or *Hvem^WT/tm^* hemizygous mice, HVEM expression by *Hvem^tm/tm^* mice was sufficient to keep CD5 and PD-1 expression at a level similar to that in WT mice (figure 4*b*,*c*, electronic supplementary material, figure S8E). This suggests it is engagement with BTLA that underlies HVEM’s influence on CD5 and PD-1 expression, not signalling through the HVEM cytoplasmic domain.

### 2.4. BTLA signalling in newly generated T cells blocks autoimmune disease

**Figure 5.**
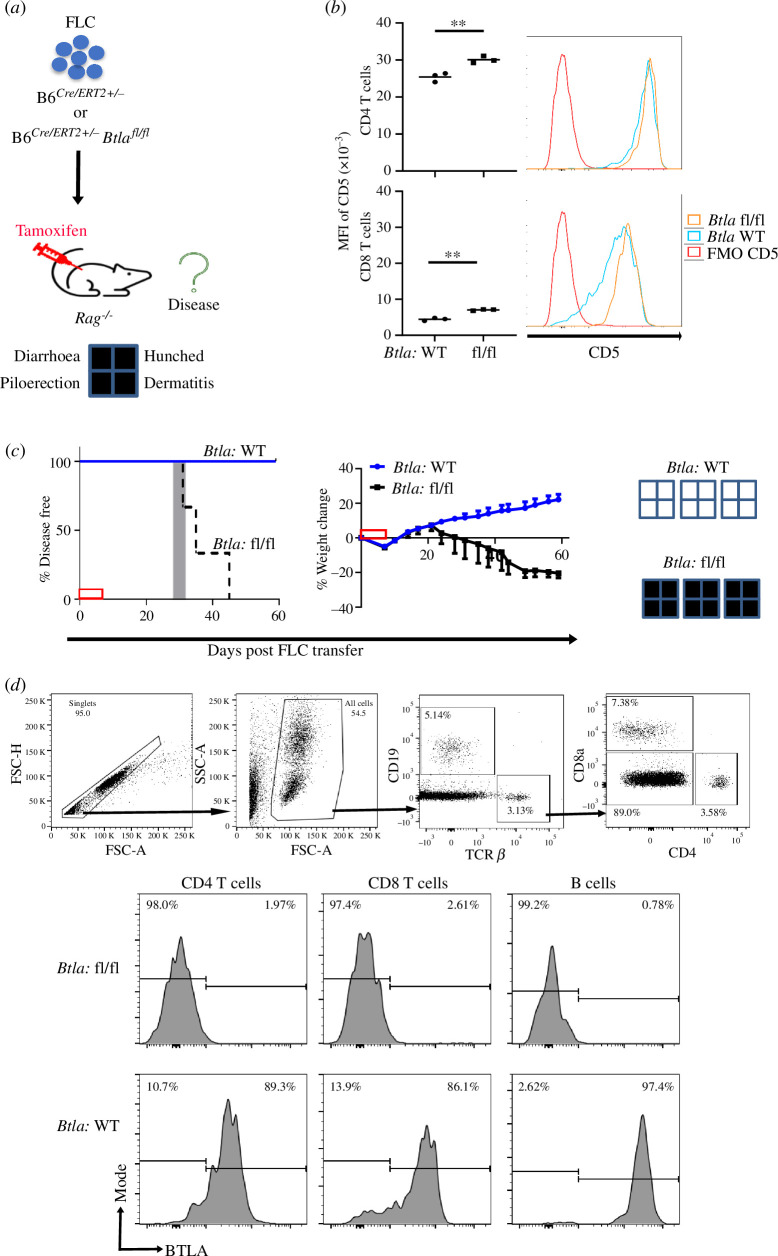
Loss of BTLA early in T cell ontogeny generates autoimmune disease. (*a*) We adoptively transferred 20 × 10^6^ FLC pooled from 8 to 10 embryonic day 14–16 B6*^Cre/ERT2+/−^* (WT) or B6*^Cre/ERT2+/−^ Btla^fl/fl^* (fl/fl) foetuses to 7 week old *Rag^–/–^* mice on day 0 (*n* = 3 recipients per group), followed by tamoxifen injection on days 0, 1, 3, 5 and 6. Recipient mice were monitored for signs of disease for eight weeks post-FLC transfer. (*b*) MFI of CD5 in CD4 T cells (top) and CD8 T cells (bottom) with respective representative histograms of peripheral T cells in the recipients of FLC from B6*^Cre/ERT2+/−^* and B6*^Cre/ERT2+/−^ Btla^fl/fl^* at eight weeks post-tamoxifen. Dots indicate data from individual mice; **p < 0.01. (*c*) Left panel: disease incidence in recipients of B6*^Cre/ERT2+/−^* (blue line) B6*^Cre/ERT2+/−^ Btla^fl/fl^* (black dashed line) FLC. Survival curves were significantly different, *p* = 0.02. The grey rectangle indicates the range, in days, at which the first T cells were detected in the peripheral blood after FLC transfer. Right panel: weight changes in recipients of B6*^Cre/ERT2+/−^ Btla^fl/fl^* FLC or B6*^Cre/ERT2+/−^* FLC. The red box on the X-axes indicates the tamoxifen treatment period. The presence (filled) or absence (empty) of disease signs is depicted on the far-right panel. (*d*) Flow cytometry gating (top). A representative histogram of BTLA expression in the T and B cells populating the periphery of B6*^Cre/ERT2+/−^ Btla^fl/fl^* FLC recipient mice (middle) or B6*^Cre/ERT2+/−^ Btla^fl/fl^* FLC recipient mice (bottom) at four weeks post-FLC transfer is shown.

Expression of BTLA and its effects on CD5 levels early in T cell ontogeny (figure 1*b*,*d*) may reflect a need for this co-inhibitor to ‘tune’ developing T cells to establish tolerance to self-peptide/MHC complexes. Previously, we showed that newly generated T cells depended on PD-1 to broadly establish self-tolerance; transfer of PD-1-deficient foetal liver cells (FLC) to syngeneic *Rag^−/−^* recipients led rapidly to a lethal multi-organ autoimmune disease as newly generated T cells emerged from the thymus [44,45]. To test if BTLA expression is similarly functionally important in newly generated T lymphocytes, we used three approaches that included either stimulating BTLA signalling or removing it. First, we treated recipients of *Pdcd1^−/−^* FLC with an agonistic antibody to BTLA. In this haematopoietic stem cell (HSC) transfer model, T cells typically begin to seed the periphery approximately three weeks post-FLC injection [44]. Shortly after T cell generation recipients of *Pdcd1^−/−^* HSC developed symptoms of disease. The agonistic anti-BTLA antibody significantly delayed disease development (electronic supplementary material, figure S9). In the two additional approaches, we adoptively transferred FLC that were either congenitally deficient in BTLA or BTLA was inducibly deleted after transfer (figure 5*a*). We transferred FLC from *B6^Cre/ERT2+/−^ Btla^fl/fl^* or *B6^Cre/ERT2+/−^* control mice to adult *Rag^−/−^* mice, followed by injection of tamoxifen to induce *Btla* gene deletion in the transferred cells. FLC was used as a source of HSC in this experiment, allowing for the deletion of BTLA in T cell progenitors pre-thymic selection. Similar to the germline *Btla^−/−^* T cells, peripheral T cells in the recipients of FLC from *B6^Cre/ERT2+/−^ Btla^fl/fl^* mice had increased CD5 expression level through the eight week experimental period (figure 5*b*). T cells were detected in the peripheral blood of FLC recipient mice around four weeks post-FLC transfer, which coincided with the onset of disease in recipients of *B6^Cre/ERT2+/−^ Btla^fl/fl^* FLC (figure 5*c*). All the recipients of *B6^Cre/ERT2+/−^ Btla^fl/fl^* FLC were diseased before the experimental endpoint while their *B6^Cre/ERT2+/−^* counterparts remained free of disease (figure 5*c*). Lymphocytes populating the periphery of recipients of *B6^Cre/ERT2+/−^ Btla^fl/fl^* FLC were BTLA negative while most of lymphocytes populating *B6^Cre/ERT2+/−^* FLC recipients expressed BTLA (figure 5*d*). Although BTLA expression has been seen on non-haematopoietic cells in some settings [69,70], these data indicated that loss of BTLA in foetal liver derived cells alone was sufficient for the generation of multi-organ autoimmune disease in the recipients.

To examine in more detail the autoimmune disease generating effects of BTLA deficiency in newly generated versus established *Btla^−/−^* T cells, we compared transfers of FLC, thymocytes, whole splenocytes or sorted splenic T cells to syngeneic *Rag^−/−^* recipients. Most of the recipients of *Btla^−/−^* thymocytes or FLC demonstrated severe morbidity, while recipients of the WT control FLC, FLC deficient in Fas (*lpr* FLC) and recipients of *Btla^−/−^* whole splenocytes or purified splenic T cells remained free of disease (electronic supplementary material, figure S10A). Histological analysis of tissue sections obtained from sick mice 60–65 days post-cell transfer revealed lymphocytic infiltrations in major organs, including the liver, kidney and pancreas of *Btla^−/−^* FLC recipients (electronic supplementary material, figure S10B). Lymphocytic infiltration was present in the liver of *Btla^−/−^* FLC but not WT FLC recipients and included CD4 and CD8 T cells (electronic supplementary material, figure S10C). The liver was the most frequently affected internal organ examined in *Rag^−/−^* recipients of *Btla^-/-^* FLC, consistent with the late life spontaneous hepatitis that has been observed in unmanipulated *Btla^-/-^* mice [32]. Newly generated *Btla^−/−^* T cells demonstrated substantially increased proliferation compared with WT T cells (electronic supplementary material, figure S10D). Collectively, these data demonstrated a requirement for BTLA in newly generated T cells to establish tolerance and prevent lymphopaenia-potentiated autoimmune disease, while BTLA was not required for maintaining tolerance of established T cells under these conditions.

### 2.5. CD4^+^ T cells and MHC II are required for autoimmune disease

**Figure 6.**
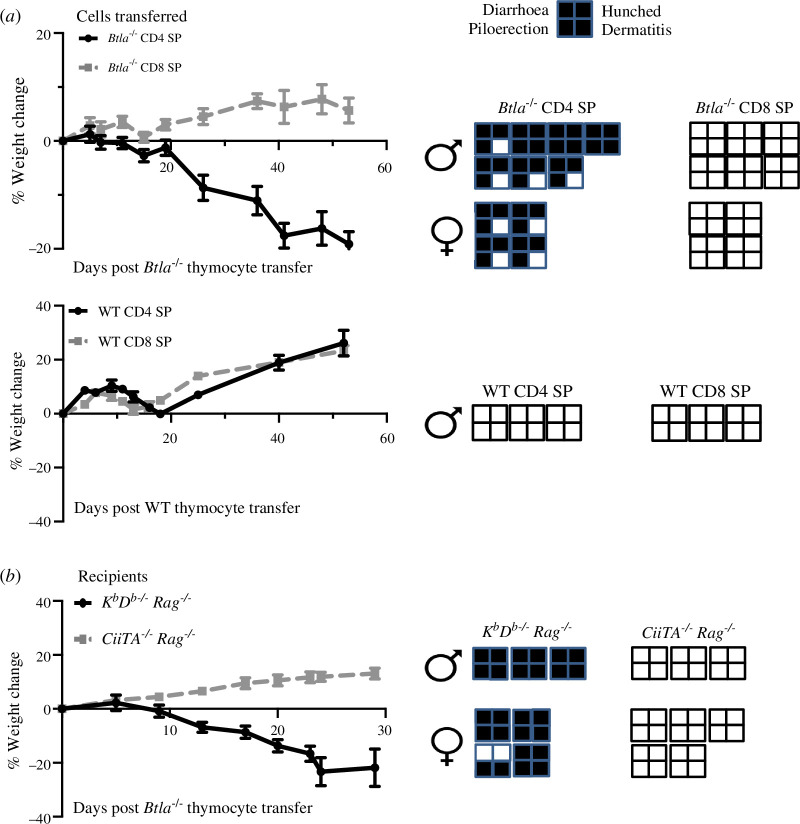
Autoimmune disease in Btla^–/–^ thymocyte recipients requires CD4^+^ T cells and MHC II. (*a*) We adoptively transferred 3 × 10^6^ MACS-sorted CD4 or CD8 SP thymocytes pooled from seven 8–10 week old B6.*Foxp3*^*EGFP*^ × *Btla*^*−/−*^ (left column) or B6.*Foxp3*^*EGFP*^ (right column) mice i.v. to 8–10 week old *Rag*^*−/−*^ mice (*Btla*^*–/–*^ thymocyte recipients, *n* = 10–11 mice/group; WT thymocyte recipients, *n* = 3 mice/group) and monitored for several weeks or after losing ≥ 20% of baseline body weight, whichever came first. Body weight change of the *Btla*^*–/–*^ SP thymocyte recipients (data were from three independent experiments) or WT SP thymocyte recipients is shown, and the presence (shaded) or absence (unshaded) of disease signs is depicted to the right of the graphs. (*b*) Thymocytes containing 3 × 10^6^ SP cells (i.e. non-sorted) pooled from seven 8–10 week old B6.*Foxp3*^*EGFP*^ × *Btla*^*−/−*^ mice were injected via tail vein to 8–10 week old *K*^*b*^*D*^*b–/–*^ *Rag*^*–/–*^ mice (*n* = 7) or *CiiTA*^*–/–*^ *Rag*^*–/–*^ (*n* = 8) mice, which were then monitored for several weeks or until after losing ≥ 20% of baseline body weight, whichever came first. Body weight change of recipient mice is shown. Data are from two independent experiments. The presence (shaded) or absence (unshaded) of disease signs is depicted to the right of the graph. Cell donors and recipients were of the sex indicated.

Having shown that loss of BTLA in newly generated T cells leads to autoaggressive T cells, we asked what T cell subset(s) is required for disease generation. We adoptively transferred sorted CD4 or CD8 SP thymocytes from *Btla**^−/−^*** or WT mice into *Rag**^−/−^*** mice (figure 6*a*, electronic supplementary material, figure S11A). We monitored the recipient mice for several weeks or until losing ≥ 20% of baseline body weight, whichever came first. Recipients of *Btla**^−/−^*** CD4 SP T cells started losing weight as early as 21 days post-cell transfer, which continued for up to 53 days when they had lost ≥ 20% of their baseline body weight (figure 6*a*). All of the *Btla**^−/−^*** CD4 SP T cell recipient mice had a hunched appearance, piloerection, diarrhoea and some had dermatitis. Disease occurred both in male and female *Rag**^−/−^*** recipients of *Btla**^−/−^*** CD4 SP T cells. By contrast, none of the recipients of *Btla**^−/−^*** CD8 SP T cells showed signs of morbidity, and their body weight increased throughout the experiment. Although recipients of WT SP CD4 or CD8 T cells had an initial decline in body weight, they quickly recovered and showed an improvement in body weight and no signs of disease (figure 6*a*). To examine what MHC molecule is required for this autoimmune disease, we adoptively transferred whole *Btla**^−/−^*** SP thymocytes to *K^b^D^b**−/−**^ Rag**^−/−^*** or *CiiTA**^−/−^** Rag**^−/−^*** mice that lacked both Rag and MHC class I genes or lacked both Rag and MHC class II protein expression [71], respectively. All *CiiTA**^−/−^** Rag**^−/−^*** mice were free of disease, while all *K^b^D^b**−/−**^ Rag**^−/−^*** recipients had signs of morbidity within 3 weeks post-T cell transfer, regardless of sex (figure 6*b*). Similarly, sorted CD4 *Btla**^−/−^*** SP thymocytes transferred to *K^b^D^b**−/−**^ Rag**^−/−^*** caused disease, while sorted CD8 *Btla**^−/−^*** SP thymocytes transferred to *CiiTA**^−/−^** Rag**^−/−^*** mice did not (electronic supplementary material, figure S11B,C). Thus, MHC class II and MHC class II-restricted CD4 T cells were required to induce disease.

### 2.6. BTLA and PD-1 signalling are needed in newly generated T cells to block autoimmune disease

**Figure 7.**
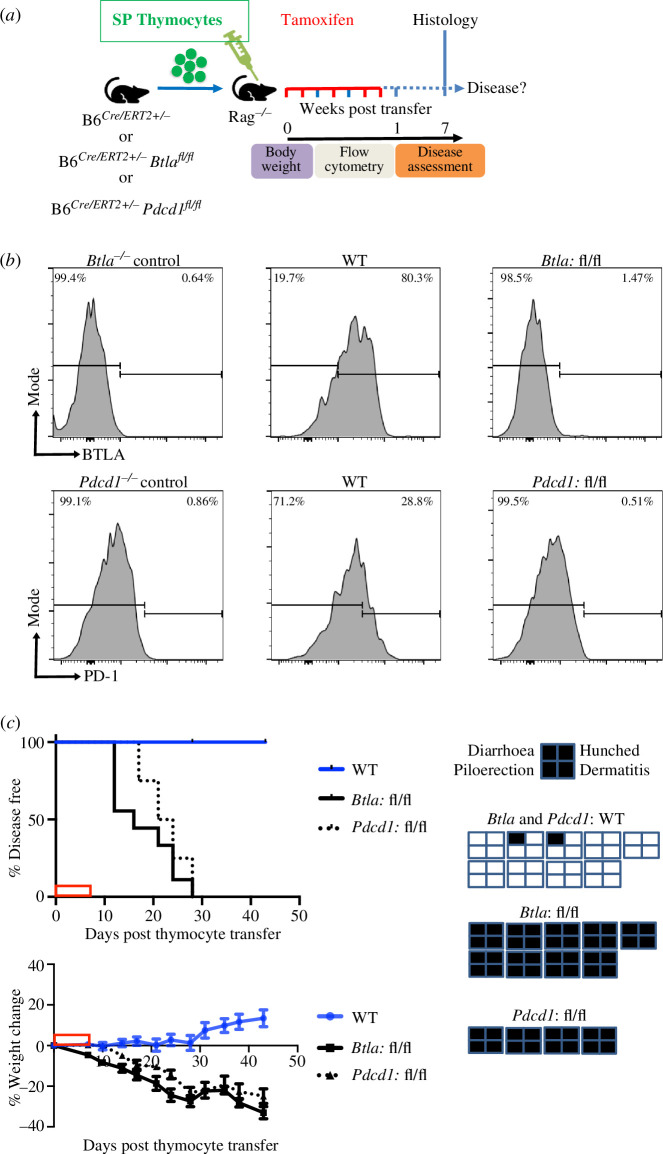
BTLA and PD1 are needed post-thymic selection in newly generated T cells to block autoimmune disease. (*a*) We adoptively transferred thymocytes containing 5 × 10^6^ SP (non-pooled) from 7 to 12 week-old B6^*Cre/ERT2+/−*^ (WT) or B6^*Cre/ERT2+/−*^ *Btla*^*fl/fl*^ (fl/fl) mice or B6^*Cre/ERT2+/−*^ *Pdcd1*^*fl/fl*^ (fl/fl) mice to 7–12 week-old *Rag*^*–/–*^ mice on day 0, followed by tamoxifen injection on days 0, 1, 3, 5 and 6. Mice were then monitored for signs of disease for seven weeks. (*b*) Flow cytometry gated on TCRβ^+^ cells. Representative histograms of BTLA (top row) or PD-1 (lower row) expression in the T cells of germline gene knockout control mice and B6^*Cre/ERT2+/−*^ or B6^*Cre/ERT2+/−*^ *Btla*^*fl/fl*^ or B6^*Cre/ERT2+/−*^ *Pdcd1*^*fl/fl*^ thymocyte recipients at two weeks post-thymocyte transfer (i.e. seven days post-tamoxifen) is shown. (*c*) Top left panel: disease incidence in recipients of B6^Cre/ERT2+/−^ (*n* = 9) or B6^*Cre/ERT2+/−*^ *Btla*^*fl/fl*^ (*n* = 9) or B6^*Cre/ERT2+/−*^ *Pdcd1*^*fl/fl*^ (*n* = 4) thymocytes. Disease-free survival curve comparison demonstrated a significant difference between both fl/fl groups and the WT, with *p* < 0.0001. Data are combined from three independent experiments (two for *Btla*: fl/fl and one for *Pdcd1*: fl/fl; WT were included in all three experiments). Bottom left panel: weight changes in the indicated of B6^*Cre/ERT2+/−*^ *Btla*^*fl/fl*^ or B6^*Cre/ERT2+/−*^ *Pdcd1*^*fl/fl*^ or B6^*Cre/ERT2+/−*^ thymocyte recipients. The presence (shaded) or absence (unshaded) of disease signs is depicted on the far-right panels. The red box on the X-axis indicates the tamoxifen treatment period.

The current studies identified that, like PD-1, BTLA was important for the establishment of tolerance in newly generated T cells; however, they did not address whether these co-inhibitory receptors were needed during thymic selection or post-thymic selection, when the newly generated T cells seed the periphery. To examine if autoimmune disease occurs when BTLA or PD-1 is deleted post-thymic selection, thymocytes from adult (7–9 week-old) *B6^Cre/ERT2+/−^ Btla^fl/fl^* or *B6^Cre/ERT2+/−^ Pdcd1^fl/fl^* mice were adoptively transferred via the tail vein to adult *Rag****^−/−^*** mice, followed by tamoxifen injection to induce gene deletion in the transferred thymocytes. The control group received *B6^CreERT2+/−^* thymocytes and tamoxifen injection (figure 7*a*). There was a near complete deletion of BTLA and PD-1 by day 7 post the last dose of tamoxifen in the T cells of *B6^Cre/ERT2+/−^ Btla^fl/fl^* and *B6^Cre/ERT2+/−^ Pdcd1^fl/fl^* thymocyte recipients, respectively, while expression of these co-inhibitors was maintained in the *B6^CreERT2+/−^* thymocyte recipients (figure 7*b*). All of the *B6^Cre/ERT2+/−^ Btla^fl/fl^* and *B6^Cre/ERT2+/−^ Pdcd1^fl/fl^* thymocyte recipients, but not their *B6^CreERT2+/−^* counterparts, developed inflammatory disease. They began to lose body weight at days 7–14 post-thymocyte transfer and showed additional signs of disease at days 12–17 (figure 7*c*). Histological examination showed CD4 SP T cell infiltration in the kidney and liver of *B6^Cre/ERT2+/−^ Btla^fl/fl^* thymocyte recipients and the kidney, liver and lung of *B6^Cre/ERT2+/−^ Pdcd1^fl/fl^* thymocyte recipients (electronic supplementary material, figure S12). Since BTLA and PD-1 were present during thymic selection and deleted only post-cell transfer, these co-inhibitors were needed at the RTE stage to establish tolerance.

## 3. Discussion

In this study, we aimed to understand the relationship between the constitutively expressed T cell co-inhibitory molecules BTLA [72,73] and CD5, and an induced co-inhibitor, PD-1, in the steady state and under conditions that promote multi-organ autoimmune disease. Our data on steady state CD5 expression in the thymus and spleen were consistent with findings in the literature [36,39] and when compared with BTLA expression, revealed an inverse relationship. Our data showed that peripheral T cells expressed higher levels of BTLA than thymocytes. This is a result of a maturation process that began in the thymus and continued in the periphery, with RTE in the spleen expressing lower levels of BTLA relative to the mature splenic T cells. By contrast, splenic RTE expressed higher levels of CD5 relative to their mature T cell counterparts, as seen previously [40]. High CD5 on RTE may reflect increased sensitivity to self-peptide–MHC complexes, consistent with the greater self-reactivity and autoimmune potential of RTE that we have observed in the context of PD-1 deficiency [45].

Analysis of CD5 expression in the *Btla^−/−^* mice or upon induced BTLA deletion showed that the overall mean CD5 expression was enhanced in BTLA-deficient CD4 and CD8 T cells, indicating that BTLA negatively regulates CD5 expression. Heightened CD5 expression was a result of a reduction in CD5 low cells. This suggests that BTLA was either needed for survival of CD5 low cells or its absence enhanced CD5 expression in the CD5 low cells. While a role for BTLA in T cell survival during chronic stimulation has been shown [74], we found increased expression of CD5 on BTLA-deficient OT-II CD4 T cells, a T cell that normally expresses relatively low CD5 levels [38]. This indicated BTLA may be able to negatively regulate CD5 expression independently from any effects it might have on the generation of the TCR repertoire early in life. CD5 expression on differentiating thymocytes reflects the TCR affinity for self-peptide−MHC complexes. Regulation of CD5 expression in thymocytes appeared to be specific to BTLA, as a deficiency in another co-inhibitor (PD-1) had no effect on CD5 expression. Complementing the CD5 expression data, the *Btla^−/−^* thymic and splenic CD8 T cells displayed enhanced Nur77^GFP^ expression. Although our data showed a disparity between CD5 and Nur77^GFP^ expression levels in the *Btla^−/−^* thymic and splenic CD4 T cells, Zinzow-Kramer *et al*. had previously reported that a broad range exists in the expression levels of CD5, Ly6C and Nur77^GFP^ *in vivo* for naive T cells even within the same TCR niche [75]. This suggests that CD5 and Nur77 can be regulated differently from each other in response to TCR signals. Heightened PD-1 expression in CD4 and CD8 SP thymocytes in mice deficient for either BTLA or HVEM further supported the concept that loss of BTLA signalling leads to enhanced self-recognition early in T cell ontogeny. Strikingly, even the decreased levels of HVEM expression, as occurred in *Hvem^tm/tm^* mice, were sufficient to negatively regulate both BTLA and PD-1. Given that HVEM interacts with several different ligands, it could not be anticipated whether HVEM deficiency would mimic BTLA deficiency in the regulation of CD5 and PD-1 expression. HVEM deficiency mimicked BTLA deficiency closely, however, for control of CD5 expression in CD4 T cells but not in CD8 T cells. This suggested the other ligands for HVEM, including LIGHT and CD160, may have differential effects on CD5. Together these findings suggest that expression of some co-inhibitors are calibrated by BTLA signals triggered through a highly sensitive engagement by HVEM, acting not as a signalling receptor but purely as a ligand or binding partner for BTLA.

Mice deficient in BTLA develop autoimmune disease only later in life [32], suggesting that either other co-inhibitors compensate for the loss of BTLA in early life, or early events take time for their consequences to become apparent. Increased CD5 expression in the *Btla^−/−^* mice suggested that CD5 may serve as a compensatory co-inhibitory receptor. Despite this, increased CD5 on newly generated T cells was not sufficient for preventing autoimmune disease following transfer to immune-deficient mice. The conditions in *Rag^-/-^* recipients, however, in terms of the extent or rapidity of lymphopaenia induced proliferation, changes in the microbiome and others, might not fully represent the conditions in perinatal immune competent mice. Compensatory co-inhibitor expression may limit T cell autoreactivity in other contexts. In cancer therapy, it can limit the efficacy of treatment [76]. BTLA blockade has been shown to limit tumour growth and improve survival in a murine model [77] and it is a target in clinical trials [78]. The effect of blocking both CD5 and BTLA have yet to be determined. Supporting our findings in the *Pdcd1*^−/−^ mice, where CD5 expression was elevated in the splenic CD8^+^ T cells, a recent report showed that blocking CD5 together with PD-1 substantially enhanced survival and tumour cell killing [79].

We examined the role of BTLA in newly generated T cells and found that BTLA, like PD-1 [44,45], was important for establishing tolerance in newly generated, polyclonal T cells. By contrast, BTLA signalling appears to be dispensable for establishing tolerance in newly generated TCR transgenic CD8 T cells that recognize a tissue-specific antigen expressed in the thymus and pancreas [80]. This might reflect a more critical role for BTLA in CD4 T cells. Consistent with this idea, we found that sorted *Btla**^−/−^*** CD4 but not CD8 T cells could generate multi-organ autoimmune disease, and disease development did not occur in MHC class II-deficient recipients. BTLA was also previously reported to regulate CD4 T cell alloreactivity and proliferative responses to MHC class II antigens [81]. CD4 T cells and/or MHC class II are also required for disease development caused by newly generated *Pdcd1**^−/−^*** T cells [44,45]. Disease occurred despite intact Treg numbers and function in the recipients of *Pdcd1**^−/−^*** RTE [47]. Whether BTLA signalling is needed in conventional and/or Treg cells to prevent the multi-organ autoimmune disease will require further studies. Although BTLA-deficient thymus and spleen did not have reduced Treg cell numbers, and intact suppressive function has been observed previously in BTLA-deficient Treg [82], we did not examine the function of the Treg.

To understand at what point during T cell development BTLA and PD-1 may be needed to establish tolerance, we deleted each of these co-inhibitors individually in RTE that had the capacity to express them during thymic development. We found that loss of BTLA or PD-1 post-thymic selection in RTE resulted in autoimmune disease in lymphopenic host mice, associated with loss of weight, dermatitis, diarrhoea and T cell infiltration of organs. Thus, BTLA and PD-1 were needed post-thymic selection to establish peripheral tolerance in newly generated T cells, at least in *Rag^−/−^* mice. Our current study does not exclude a role for these co-inhibitors during central tolerance. A role for BTLA in central tolerance has not yet been examined. We previously found that PD-1 was not needed for central tolerance in CD4 or CD8 T cells specific to a ubiquitous self-antigen [44], however, PD-1 was needed for non-deletional central tolerance in CD8 T cells that recognize a tissue-restricted antigen [80]. The current data suggest that any role the co-inhibitors might have in inducing central tolerance is not by itself sufficient to broadly establish a durable self-tolerance. The need for these co-inhibitors in RTE suggests that BTLA and PD-1 will be critical to establishing tolerance during the neonatal period when all peripheral T cells are RTE [83,84]. Consistent with this idea, a substantial proportion of neonatal CD4 T cells, those undergoing natural lymphopaenia-induced proliferation, were found to express PD-1 [45]. Our current studies are further addressing this important question.

Interestingly, adult mouse splenic T cells from mice with germline BTLA or PD-1 [44] deficiency did not cause overt autoimmune diseases in the recipients. From *Pdcd1**^−/−^*** splenocytes, only the sorted RTE subpopulation could generate overt disease; sorted established T cells did not cause disease [45]. A key question for future studies is whether the seeming lack of need for these co-inhibitors in established T cells indicates that the co-inhibitors are not required to maintain a tolerant state to tonic self-peptide/MHC ligands. An alternative possibility is that these co-inhibitors are needed to maintain tolerance when T cells have developed in their presence. Although our study establishes the importance of BTLA and PD-1 signalling in RTE to block autoimmune disease development in lymphopenic recipients, the germline BTLA or PD-1 knockout mice seem to be protected from overt autoimmune disease during the neonatal period when there is physiological lymphopaenia. This is likely to result from two factors: (i) their T cell repertoire is first generated early in life, a period naturally deficient in lymph node stroma [85], and (ii) early disease-causing events normally take time for their consequences to become apparent. Consistent with the first idea, we previously showed that neonatal *Rag**^−/−^*** recipients of *Pdcd1**^−/−^*** FLC were relatively resistant to disease, as were adult recipients that lacked or had reduced lymph nodes [44]. Supporting the second idea, T cells generated early in life in NOD mice play an important role in the initiation of insulitis long before the onset of overt diabetes that manifests much later in life [86,87].

Taken together, our data identify a regulatory axis between BTLA and CD5 and a critical need for both constitutive and inducible co-inhibitors (BTLA and PD-1) when T cells initially seed the periphery.

## 4. Material and methods

### 4.1. Mice

Mice used in this study included male and female B6.129S7-*Rag1*^tm1Mom^/J (Rag^−/−^), B6.Cg-*Foxp3*^tm2(EGFP)Tch^/J (*Foxp3^EGFP^*) [88,89], B6.129-Gt(ROSA)26Sor^tm1(cre/ERT2)Tyj^/J (B6*^Cre/ERT2+^*), C57BL/6J (H-2^b^; B6), B6.129S2-Ciita^tm1Ccum^/J (*CiiTA^−/−^*) and B6.MRL-*Tnfrsf6^lpr^*/J (*lpr*) mice were originally purchased from the Jackson Laboratory (Bar Harbor, ME, USA). The C57BL/6 H-2K^btm1^-H-2D^btm1^N12 (*K^b^D^b**−/−**^*) were originally obtained from the National Institute of Allergy and Infectious Diseases (NIAID) Exchange Program (NIH: 004215) [90]. We generated the *CiiTA**^−/−^** Rag**^−/−^*** mice and *K^b^D^b**−/−**^ Rag**^−/−^*** mice by crossing the *Rag^−/−^* mice with *CiiTA**^−/−^*** mice and *K^b^D^b**−/−**^* mice, respectively. B6.*Rag2p^GFP^* mice [42,43] were kindly provided by Dr Pamela Fink (University of Washington, Seattle, WA), and we previously generated B6.*Rag2p^GFP^ Pdcd1^−/−^* mice [45]. C57BL/6-*Btla^−/−^* (abbreviated as *Btla^−/−^*) were originally provided by Dr Kenneth Murphy (Washington University, St. Louis, MO), and C57BL/6-*Pdcd1^−/−^* (abbreviated as *Pdcd1^−/−^*; backcrossed 11 generations to C57BL/6; originally generated by Prof. T. Honjo and colleagues [91]) mice were bred at the University of Alberta. We crossed OT-II mice (kindly provided by Dr Sue Tsai, University of Alberta) and OT-I mice (kindly provided by Dr M. McGargill, St. Jude Children’s Research Hospital, Memphis, TN) to *Btla^−/−^* mice, and crossed *Foxp3^EGFP^* mice to *Btla^−/−^* and *Pdcd1^−/−^* mice [47]. The B6.*Pdcd1^fl/fl^* mice were purchased from Taconic Biosciences (Rensselaer, NY, USA). B6.*Btla^fl/fl^* mice [33,92] generated by us (M.K.) and were kindly provided by John R. Šedý and Carl Ware (Sanford Burnham Prebys Medical Discovery Institute, La Jolla, USA). B6.*Nur77^GFP^* mice [54] were provided by Dr Kristin Hogquist (University of Minnesota, Minneapolis, MN) and we generated *Btla^−/−^ Nur77^GFP^* mice. *Hvem^−/−^*, *Hvem^fl/fl^* and mice with the cytoplasmic tail of *Hvem* deleted mice were bred and analysed at La Jolla Institute for Immunology, La Jolla, CA, USA. All mice were between 7 and 24 weeks old. Animal care was in accordance with the Canadian Council on Animal Care guidelines. The studies were performed under Animal Use Protocol 00000215, approved by the Animal Care and Use Committee Health Sciences of the University of Alberta. Mice were housed under clean conventional housing conditions at the University of Alberta Health Sciences Laboratory Animal Services (HSLAS) facilities. Mouse studies carried out at La Jolla Institute for Immunology used animals bred and housed under specific pathogen-free conditions and were approved by the La Jolla institute for Immunology Animal Care and Use Committee under protocol AP00001007.

### 4.2. Generation of HVEM mutant mice

Mouse HVEM cytoplasmic tail mutant (*Hvem^tm^*) mice were generated by the CRISPR/Cas9 system by injection of a sgRNA–Cas9 complex plus a donor specific single-stranded DNA (ssDNA) into C57BL/6 pronuclear embryos. All materials for the CRISPR/Cas9 system were purchased from Integrated DNA Technologies (IDT, Newark, NJ). The specific sgRNA targeted the front of the *Tnfrsf14* exon 7: 5′-AGAACAUCAAGUCAUGGGAG-3′. The ssDNA homology directed repair (HDR) template has a stop codon in the exon 7 of the *Tnfrsf14* locus: 5′-CCATAAGCATATGCCAGTTGGAACTTCCTCCCCGACCCAGTTATACCTGGAAAGG CTCCAGCTCCTTAGTCACTTAGCCTGTAACACAAGAACATCAAGTCATGGGAGAGCT

GAAGCAAGAGGGGAGGGAGACGGGCACACAGCAATGAAAAACCCACATTCTGGGATTCCAGCTGTGTGATCTACCTCCCAAGTCTGAC-3′. The HDR repair did not occur, but we obtained an F0 founder that has an allele with a deletion of the whole exon 7 and a read-through out-of-frame amino acid sequence by splicing exon 6 to exon 8. The F0 founder was backcrossed to the WT C57BL/6 strain for at least two generations. We obtained homozygous offspring (*Hvem^WT^* and *Hvem^tm^*) by intercrossing the N2 generation of *Hvem^+/tm^* mice. The mice were generated at the RIKEN Center for Integrative Medical Sciences (IMS), Yokohama, Japan. All procedures related to strain construction were approved by the RIKEN IMS Animal Care and Use Committee. We confirmed that the tail-mutated HVEM protein was incapable of signalling *in vitro* and *in vivo*, as described previously [67]. For *in vitro* testing, the pGL4.32[luc2P/NF-κB-RE/Hygro] (NF-κB-driven firefly luciferase; Promega) and pRL-TK (*Renilla* luciferase as an internal control; Promega) plasmids were co-transfected with mouse *Hvem^WT^*, or the *Hvem^tm^* sequence with deletion of exon 7, or control vector (EGFP) plasmids into 293 T cells by TransIT-LT1 (Mirus). One day later, transfected cells were co-cultured with transfected 293 T cells expressing mouse LIGHT, CD160, or BTLA. On the next day, firefly and *Renilla* luciferase activity were measured through the Dual-Glo Luciferase Assay System (Promega) and detected by the EnVision 2104 Multimode Plate Reader (PerkinElmer). For *in vivo* testing, we used a hepatic injury model. Co-housed female littermates were injected with 2 µg αGalCer (KRN7000; Kyowa Kirin Research) in a total volume of 200 µl PBS by retro-orbital injection. Serum ALT activity was measured using a colorimetric/fluorometric assay kit (ab105134; Abcam) at 24 h after injection as described previously [67].

### 4.3. Cell transfer, tamoxifen-induced conditional deletion and disease criteria

Thymocytes or splenocytes containing the indicated number of SP T cells were injected into adult *Rag^−/−^* mice. Briefly, thymuses and spleens were removed from the donors and mashed with glass slides on ice to make a single-cell suspension, followed by filtration with a 70 µm cell strainer (Fisherbrand™). CD4 and CD8 SP T cells were isolated by negative selection from a single cell thymocyte suspension using EasySep™ Mouse naive CD4^+^ T Cell Isolation Kit (#19765) and EasySep™ Mouse naive CD8^+^ T Cell Isolation Kit (#19858), respectively, from STEMCELL Technologies (Vancouver, Canada) according to manufacturer’s instructions. The purity of the sorted cell population was >96%. Viability was assessed by trypan blue exclusion and >90% viable cells were used for experiments. Where indicated, TCR^+^ CD24^low^ cells were sorted from splenocytes of six-week-old *Btla^−/−^* mice on a FACS BD influx™ cell sorter (BD Biosciences). The purity of the sorted cell populations was 92%. Foetal liver cells (FLC) were used as a source of haematopoietic stem cells (HSC) and were harvested from embryonic day 14–16 foetuses. A single-cell suspension was made on ice by gently pipetting the foetal livers and filtration through a 70 µm cell strainer (Fisherbrand™). Viability was assessed by trypan blue exclusion and >90% viable cells were used for experiments. Six−eight-week-old male and female *Rag^−/−^* mice were used as recipients, and each recipient received 10–20 × 10^6^ FLC, followed by tamoxifen injection as described below. Some HSC recipients also received agonist anti-BTLA antibody (6A6; 10 μg/g body weight) or hamster IgG isotype control antibody once per week beginning 18 days post-foetal liver injection. To induce BTLA or PD-1 deletion, B6*^Cre/ERT2+/−^ Btla^fl/fl^* mice or adult *Rag^−/−^* recipients of B6*^Cre/ERT2+/−^ Btla^fl/fl^* or B6*^Cre/ERT2+/−^ Pdcd1^fl/fl^* FLC or thymocytes were intraperitoneally injected with 1.4 mg tamoxifen (Sigma-Aldrich) in corn oil (+5% (vol/vol) ethanol) on days 0, 1, 3, 5 and 6. Recipients of control B6*^CreERT2+/−^* FLC or thymocytes also received tamoxifen injection. Signs of disease included loss of weight, hunched appearance, piloerection, diarrhoea and dermatitis. Recipient mice were no longer considered disease-free when two or more of the above signs were evident, or mice had lost ≥20% of baseline body weight. In addition, for mice to be classified as diseased, disease signs must persist for at least two weeks. Immunohistochemistry staining was performed on tissues from multiple organs collected from recipient mice.

### 4.4. Immunohistochemistry

Mice were euthanized and transcardially perfused with phosphate buffer saline (PBS), followed by 4% paraformaldehyde (PFA) in PBS. Harvested non-lymphoid organs (heart, kidneys, liver and lungs) were immersed in 4% PFA in PBS overnight at 4°C and then transferred into fresh 30% sucrose in PBS every day for two consecutive days at 4°C. Tissues were embedded in optimum cutting temperature compound (TissueTek OCT, Sakura Finetek, 4583), frozen on liquid nitrogen and cryosectioned (Leica CM1950) at −20°C with a thickness of 5 or 10 µM on glass slides. Following three washes in 1× PBS, tissue sections were blocked in 10% normal goat serum for 1 h at room temperature and incubated in rat anti-mouse CD4 (1:200; MCA2691; Bio-Rad) or CD8a (Biolegend, San Diego, CA) antibody overnight at 4°C. Slides were washed in PBS-Tween (0.5% Tween 20 in 1× PBS) and incubated in goat anti-rat IgG Alexa Fluor^®^ 488 (1:200; A11006; Life Technologies) antibody for 45 min at room temperature. To visualize cellular nuclei, tissue sections were counterstained with VECTASHIELD mounting medium with DAPI (Vector Laboratories, H-1200). Spleens from *Rag^−/−^* and WT mice were used as the CD4 negative and positive control, respectively. The negative control for primary antibody specificity was omitting the primary antibody in the staining. Immunofluorescence images were taken using either a Leica DM IRB Microscope and Open Lab software or an Axioplan, Axiovision 4.1 software (Carl Zeiss, Toronto, ON). An average of three images per section were examined.

### 4.5. Flow cytometry and BrdU incorporation

Fluorochrome conjugated antibodies for flow cytometry staining used in this study were purchased from ThermoFisher Scientific: murine anti-TCRβ (H57-597), CD4 (RM4-5), CD5 (53-7.3), CD44 (IM7), CD62L (MEL-14), FoxP3 (FJK-16s), BTLA (6F7), PD-1 (J43); or BioLegend: CD19 (6D5), CD8α (53-6.7), HVEM (HMHV-1B18). GFP expression was also analysed in mice expressing the GFP transgene. Peripheral blood samples, thymocytes and splenocytes were stained after incubation with FcR block, which was a cocktail of anti-CD16/32 antibody (2.4G2; Bio Express, West Lebanon, NH) and mouse, rat and hamster sera. Staining was done at 4°C for 20 min, followed by washing and resuspension in Hanks’ Balanced Salt Solution (HBSS) supplemented with 2% foetal bovine serum (FBS). A BD LSR II (BD Biosciences) with FlowJo software was used for data acquisition and analyses. To assess proliferation *in vivo*, experimental mice were treated with 2 mg BrdU in PBS by i.p. injection. BrdU incorporation was assessed in splenic T cells 24 h after injection using a BrdU flow cytometry kit (BD PharMingen™).

### 4.6. TCR repertoire analysis by single-cell RNA sequencing

CD4 SP T cells were isolated by negative selection using the EasySep™ Mouse naive CD4+T Cell Isolation Kit (#19765, STEMCELL Technologies, Vancouver, Canada) according to the manufacturer’s instructions, from single-cell thymocyte suspensions prepared from B6.*Btla* WT and B6.*Btla^-/-^* mice (aged 8–12 weeks). TCR sequencing was conducted using the 10× Genomics V(D)J workflow. Briefly, single-cell TCR libraries were prepared using the Chromium Single Cell Mouse TCR Amplification Kit (Catalog #1000254). Libraries were pooled to achieve desired quantities for appropriate sequencing depths, as recommended by 10× Genomics, and were sequenced on an Illumina NovaSeq 6000 (v1.5) instrument. Alignment of reads was performed using the prebuilt Cell Ranger v7.2.0 mouse reference GRCm38 v7.0.0. Reads mapping and contig annotations were conducted using the 10× Genomics Cell Ranger 7.2.0 vdj pipeline via 10× Genomics Cloud Analysis (https://www.10xgenomics.com/, accessed 10 April 2024). Across samples of WT and *Btla^−/^*^−^, a total of 4040 and 5077 T cells were sequenced, respectively.

Single-cell TCR repertoires were analysed using scRepertoire package v2.0.0 [93] within the R environment (v4.3.1). The analysis included the examination of V gene usage, CDR3 amino acid (AA) length and diversity along the residues of the CDR3 AA sequence, based on a Shannon score. For V gene usage we plotted the subgroup of TCRAV and TCRBV genes [94,95] for a better visualization. Shared and unique clonotypes, defined by their V(D)J gene usage, in WT and *Btla^−/−^* mice were depicted by Venn diagrams. The top 20 clones from each sample were visualized using alluvial plots. Clones were represented as stacked bins, with their height indicating their frequency in the sample. Shared clones between samples are linked.

Acknowledgement: Libraries were created at the Advanced Cell Exploration Core at the University of Alberta Faculty of Medicine & Dentistry, RRID:SCR_019182, which receives financial support from the Faculty of Medicine & Dentistry, the Li Ka Shing Institute of Virology, Striving for Pandemic Preparedness—The Alberta Research Consortium and Canada Foundation for Innovation (CFI) awards to contributing investigators. Sequencing was done at the University of Calgary’s Centre for Health Genomics and Informatics.

### 4.7. Statistical analysis

Statistical analysis was performed using GraphPad Prism software. Data of biological replicates are depicted as mean ± standard error. Data were statistically analysed using Student’s *t*‐test with Welch’s correction or Wilcoxon matched-pairs signed rank test or Mann–Whitney test. Statistical analysis in experiments with one variable and three groups was performed using one-way ANOVA with Dunn’s multiple comparisons test or Kruskal–Wallis test (*p < 0.05; **p < 0.01; ***p < 0.001; ****p < 0.0001). Disease onset/incidence was compared by the Kaplan–Meier method. Probability values reported for survival curve comparisons were calculated using the Mantel–Cox method.

### 4.8. Resources

**Table:** 

resource	source	identifier
antibodies		
Alexa Fluor 488 Goat anti-rat IgG	Life Technologies	A11006
APC-eFluor 780 Anti-TCRβ clone H57−597	ThermoFisher	47-5961-82
Alexa Fluor 700 Anti-CD4 clone RM4-5	ThermoFisher	56-0042-82
PerCP-Cyanine5.5 Anti-CD5 clone 53-7.3	ThermoFisher	45-0051-82
APC Anti-FoxP3 clone FJK-16s	ThermoFisher	17-5773-82
PerCP-Cyanine5.5 Anti-CD62L clone MEL-4	ThermoFisher	45-0621-82
PE-Cyanine7 Anti-Mouse CD44 clone IM7	ThermoFisher	25-0441-82
PE Anti-BTLA clone 6F7	ThermoFisher	12-5950-82
APC Anti-PD−1 clone J43	ThermoFisher	17-9985-82
PE Anti-TCR-Vß5 clone MR9-4	BD Pharmingen	5 53 190
eFluor 450 Anti-TCR-Vα2 clone B20.1	eBioscience	48-5812-82
Super Bright 600 Anti-Mouse CD8a clone 53-6.7	ThermoFisher	63-0081-82
Brilliant Violet 421 Anti-CD19 clone 6D5	BioLegend	115537
PE Anti-HVEM clone HMHV-1B18	BioLegend	136304
Anti-CD16/32 antibody clone 2.4G2	Bioxcell	BE0307
Rat anti-Mouse CD8α clone 53-6.7	Biolegend	100701
Rat anti-Mouse CD4	Bio-Rad	MCA2691
Purified anti-BTLA clone 6A6	Bioxcell	BE0132
EasySep Mouse naive CD8^+^ T Cell Isolation Kit	STEMCELL Tech.	19858
EasySep Mouse naive CD4^+^ T Cell Isolation Kit	STEMCELL Tech.	19765
BrdU flow cytometry kit	BD PharMingen	552598
chemicals, peptides and recombinant proteins		
tamoxifen	Sigma-Aldrich	T5648-1G
TissueTek OCT	Sakura Finetek	4583
DAPI	Vector Laboratories	H-1200
αGalCer	Kyowa Kirin Research	KRN7000
critical commercial assays		
pGL4.32 NF-κB-driven firefly luciferase	Promega	E8491
Dual-Glo Luciferase Assay System	Promega	E2940
Serum ALT colorimetric/fluorometric kit	Abcam	ab105134
Chromium Single Cell Mouse TCR Amplification Kit	10× Genomics	1000254
deposited data		
TCR scRNA Sequencing Data	this paper; GEO accession	GSE269212
experimental models: cell lines		
293 T cells	ATCC	CRL-3216
experimental models: organisms/strains		
B6.129S7-*Rag1*^tm1Mom^/J	The Jackson Laboratory	JAX:002216
B6.Cg-*Foxp3*^tm2(EGFP)Tch^/J	The Jackson Laboratory	JAX:006772
B6.129-Gt(ROSA)26Sor^tm1(cre/ERT2)Tyj^/J	The Jackson Laboratory	JAX:008463
C57BL/6J	The Jackson Laboratory	JAX:000664
B6.129S2-Ciita^tm1Ccum^/J	The Jackson Laboratory	JAX:003239
B6.MRL-*Tnfrsf6^lpr^*/J	The Jackson Laboratory	JAX:000482
H-2K^btm1^-H-2D^btm1^N12	NIAID Exchange Program [90]	NIH: 004215
B6.*Btla^fl/fl^* mice	Mitchell Kronenberg [33,92]	N/A
*Hvem^−/−^*	Mitchell Kronenberg [61]	N/A
*Hvem^fl/fl^*	Mitchell Kronenberg [92]	N/A
*Hvem^tm^*	this paper	N/A
B6.*Nur77^GFP^*	Kristin Hogquist [54]	N/A
B6.*Rag2p^GFP^*	Pamela Fink [42,43]	N/A
C57BL/6-*Btla^−/−^*	Kenneth Murphy [8]	N/A
C57BL/6-*Pdcd1^−/−^*	Tasuko Honjo [91]	N/A
OT-II	Sue Tsai [55]	N/A
OT-I	Maureen McGargill [56]	N/A
B6.*Pdcd1^fl/fl^*	Taconic Biosciences	13976
oligonucleotides		
sgRNA target front of the *Tnfrsf14* exon 7	Integrated DNA Technologies	N/A
recombinant DNA		
ssDNA	Integrated DNA Technologies	N/A
software and algorithms		
Cell Ranger v7.2.0 mouse reference GRCm38 v7.0.0	10× Genomics	N/A
scRepertoire package v2.0.0	MIT	doi:10.12688/f1000research.22139.2
FlowJo software	Tree Star, Ashland, OR	N/A
GraphPad Prism software v 10	Graphpad Software, San Diego, CA	N/A
Axiovision 4.1 software	Carl Zeiss, Toronto, ON	N/A
other		
Library creation	University of Alberta	RRID:SCR_019182

## Data Availability

Data reported in this paper are available upon request from the lead contact. This paper does not report original code. Any additional information required to reanalyse the data reported in this work paper is available from the lead contact upon request. TCR scRNA Sequencing Data GEO accession: GSE269212 (https://www.ncbi.nlm.nih.gov/geo/query/acc.cgi?acc=GSE269212). Additional data supporting this article have been uploaded online as part of the supplementary material. Materials availability: the HVEM mutant mouse (Hvemtm) is available from M.K. Supplementary material is available online [96].
